# Magnetic resonance spectroscopy (MRS) of vertebral column – an additional tool for evaluation of aggressiveness of vertebral haemangioma like lesion

**DOI:** 10.2478/raon-2013-0013

**Published:** 2014-04-25

**Authors:** Miran Jeromel, Janez Podobnik

**Affiliations:** Clinical Institute of Radiology, University Medical Centre Ljubljana, Ljubljana, Slovenia

**Keywords:** aggressive vertebral haemangioma, magnetic resonance spectroscopy, percutaneous vertebroplasty

## Abstract

**Background:**

Most vertebral haemangioma are asymptomatic and discovered incidentally. Sometimes the symptomatic lesions present with radiological signs of aggressiveness and their appearance resemble other aggressive lesions (*e.g*. solitary plasmacytoma).

**Case report.:**

We present a patient with large symptomatic aggressive haemangioma like lesion in 12^th^ thoracic vertebra in which a magnetic resonance spectroscopy (MRS) was used to analyse fat content within the lesion. The lesion in affected vertebrae showed low fat content with 33% of fat fraction (%FF). The fat content in non-affected (1^st^ lumbar) vertebra was as expected for patient’s age (68%). Based on MRS data, the lesion was characterized as an aggressive haemangioma. The diagnosis was confirmed with biopsy, performed during the treatment – percutaneous vertebroplasty.

**Conclusions:**

The presented case shows that MRS can be used as an additional tool for evaluation of aggressiveness of vertebral haemangioma like lesions.

## Introduction

Vascular lesions of the musculoskeletal system are relatively common. Haemangioma is by far the most common benign tumour of the axial skeleton, occurring in 11% of spines at autopsy.^1^,^2^ The histological pattern of osseous haemangioma is characterized by the proliferation of anomalous thin – walled blood vessels and sinuses lined by endothelium between the thickened, vertically oriented trabeculae of bone.^3^,^4^

Most vertebral haemangioma (VH) are asymptomatic and discovered incidentally. However, the lesions sometimes present with radiological signs of aggressiveness, clinically manifested as local pain and neurologic deficit. Symptomatic VH occur in 0.9 to 1.2% of patients.^5^,^6^ The term aggressive or active haemangioma is used for those lesions. The differentiation of aggressive VH lesions from some other tumour lesions of vertebral column could be challenging.

Imaging characteristics of aggressive VH are: occupancy of the entire vertebral body, extension into the neural arch, expansion of osseous margins, and presence of a soft tissue component.^7^ The lesions typically contain less fat and more vascular stroma thereby producing a low signal on T1 weighted magnetic resonance (MR) images.^4^,^5^,^8^ Marked post-contrast enhancement is another characteristic. However, the lesion can resemble a solitary vertebral plasmacytoma on computer tomography (CT), as well as on MR images. Epitheloid haemangioen-dothelioma is another although rare vertebral lesion that must be encountered in the differential diagnosis.

MR spectroscopy (MRS) as advanced MR imaging modality has the potential to quantify vertebral tissue components (*i.e*. bone and marrow). Data about vertebral fat content in healthy individuals were published previously.^9^,^10^ Spectral analysis of fat content in VH is useful as it can clear out the diagnosis as well as aggressiveness of the lesion. On the basis of MRS data, the lesions that need to be treated could be selected.

Authors present a case report of a patient with symptomatic aggressive haemangioma like lesion in whom a MRS was used to analyse fat content. On the basis of MRS, the decision for biopsy and early treatment with the goal of stabilization (percutaneous vertebroplasty) was made.

## Case report

**FIGURE 1.**
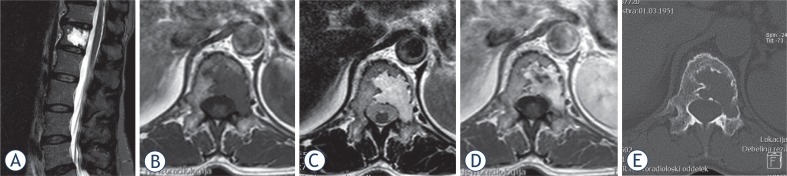
MR and CT images of the patient. The pathologic lesion in 12^th^ thoracic vertebra that fills the majority of the vertebral body is seen as a hyperintense lesion on sagittal fat suppressed (STIR) MR image (A). The lesion appeared homogeneously hypointense on pre-contrast axial *T1 w* SE MR image (B) and homogeneously hyperintense on axial *T2 w* TSE MR image (C). Post-contrast axial *T1 w* SE MR image shows marked enhancement of the lesion that extends to the left pedicle (D). The aggressiveness of the lesion was confirmed with subtle signs of cortical bone disruption seen on CT (E). However, the lesion lacked typical thickened trabeculae what brought a diagnosis of haemangioma into a question.

A 61 year old otherwise healthy female patient presented with progressive low back pain. MR examination revealed pathologic lesion in 12^th^ thoracic vertebra. The lesion filled majority of the vertebral body with extension into a left pedicle. Signal characteristics of the lesion were as follows: hyperintense on fat suppressed (STIR) MR image, homogeneously hypointense on pre-contrast *T1 w* SE MR image, homogeneously hyperintense on *T2 w* TSE MR image and very hyperintense on post-contrast *T1 w* SE MR image. Subtle disruption of the posterior vertebral wall was seen on pre-contrast MR scan. Post-contrast images confirmed homogenously enhancing (vascularized) haemangioma like tumour. The aggressiveness of the lesion was confirmed with signs of cortical bone disruption seen on CT (Figure 1).

However, the lesion lacked typical thickened trabeculae what brought a diagnosis of haemangioma into a question. A “mini brain” pattern that was partially recognized on CT raised a possibility of a plasmacytoma in the differential diagnosis. The PET scan showed no activity of the lesion. Therefore clear distinction between possible plasmacytoma was not possible with standard CT and MR imaging.

Additional MR examination with MRS was used to define fat content in order to clear out the diagnosis and confirm the aggressiveness of the lesion.

The examination was performed on Philips Achieva 1.5 T NOVA 16 channel MR scanner equipped with High Performed gradients with peak amplitude of 33 mT/m and slew rate 180 mT/m/ms. The SENSE 15 elements phased array Spine coil was used. MR imaging protocol consists of *T2 w* TSE, *T1 w* SE and STIR in sagittal plane, *T2 w* TSE, *T1 w* TSE in axial plane. After the use of the gadolinium contrast media *T1 w* sequences in sagittal and axial plane were repeated. Single Voxel (SV) spectroscopy data were acquired with STEAM (Stimulated Echo Acquisition Mode) sequence because it has several advantages compared to PRESS (Point Resolved Spectroscopy) in spine region.^10^ Parameters of SV STEAM sequences were: TR 2000 ms, TE 8.7 ms, TM (Mixing Time) 12 ms, voxel size of 19 mm × 26 mm × 17 mm, NSA (Number of Signal Averaging) 128. Water suppression was disabled and to define optimal cubic target volume PB (Pencil Beam) volume shimming was utilized. To suppress unwanted signals from adjacent structures three REST (REgional Saturation Technique) slabs were applied. The voxel of first measurement was placed in the lesion (12^th^ thoracic vertebral body) and the second in lower healthy (1^st^ lumbar) vertebral body. The data were processed on a Philips – Extended MR WorkSpace with SpectroView software. Special script for processing data was formed; only peaks from water and lipids were selected.

Peak lipid-water ratio (LWR), calculated as lipid peak/water peak was derived for each voxel. Another parameter – percent fat fraction (%FF) was used. %FF was derived from LWR as follows: LWR/(LWR+1) × 100.^10^

**FIGURE 2.**
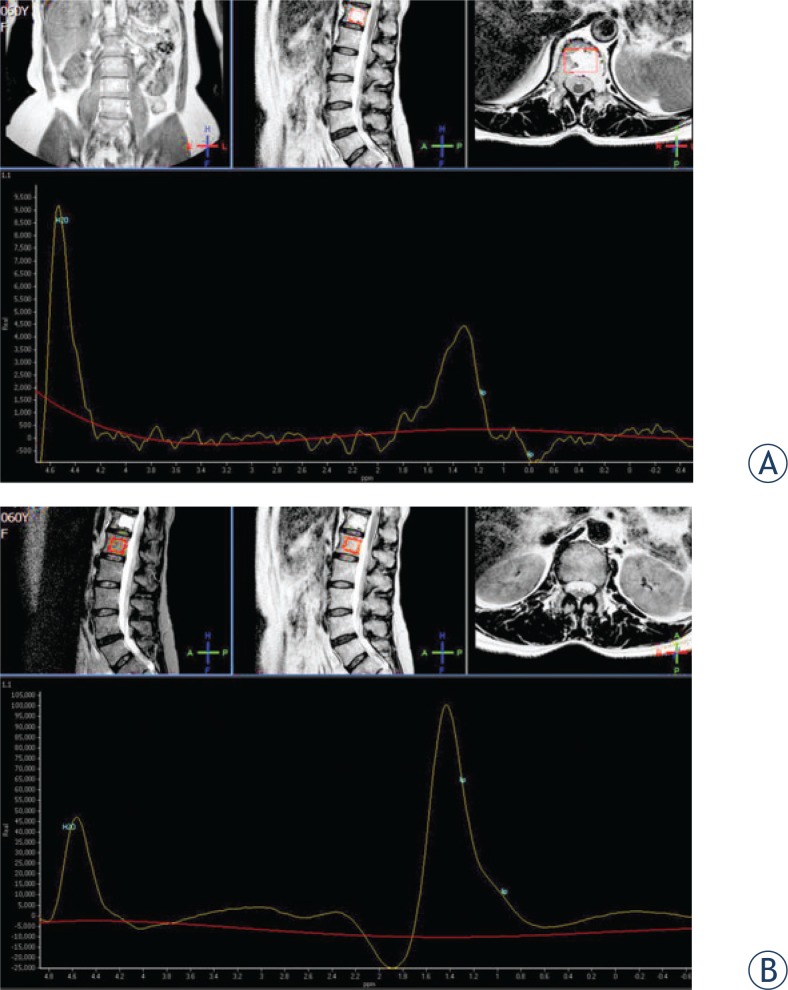
MR spectroscopy of the affected (12^th^ thoracic) and adjacent healthy (1^st^ lumbar) vertebra. Two major signals (water and lipid) separated by 3.1 ppm are clearly seen in both specters. The lipid peak in pathologic lesion (A) is much lower than in non-affected vertebral body (B). Lipid-water ratio (LWR), calculated as lipid peak/water peak in affected and adjacent vertebral body were 0.49 and 2.15, respectively. The fat content, expressed as percentage fat fraction (%FF, derived from LWR/ (LWR +1) × 100) was lower in affected (33 %FF) than in normal adjacent vertebra (68 %FF). On the basis of fat content we speculated that the amount of fat could be more a feature of aggressive haemangioma.

MRS was successfully performed in both (12^th^ thoracic and 1^st^ lumbar) vertebral bodies. Two major signals (water and lipid) separated by 3.1 ppm were identified in both spectres. A difference between lipid peaks in both vertebral bodies was noticed, as expected (Figure 2). Peak LWR in affected 12^th^ thoracic and adjacent 1^st^ lumbar vertebral body were 0.49 and 2.15, respectively. The lesion in affected vertebra showed lower fat content (33%FF) than normal adjacent vertebra (68%FF). On the basis of % FF we speculated that the amount of fat could be more a feature of haemangioma than plasmacytoma.

Based on MRS data, the lesion was therefore characterized as an aggressive haemangioma.

The diagnosis of haemangioma was confirmed with biopsy, performed during the therapeutic procedure - percutaneous vertebroplasty.

## Discussion

Vertebral haemangioma are common lesions and usually considered benign.

Both, CT and MR are important imaging modalities to help differentiate between benign and malignant lesions.^11^,^12^ It is also used to evaluate VH. The lesion shows typical pattern on CT. Multiple dots (polka-dot appearance) represent a cross-section of reinforced trabeculae.^13^ The presence of high signal intensity on T1- and T2-weighted MR images is related to the amount of adipocytes or vessels and interstitial oedema, respectively.^14^ Fatty VH may represent inactive forms of this lesion, whereas low signal intensity at MR imaging (less fat content) may indicate more active lesion with the potential to compress the spinal cord.^4^,^5^,^8^,^13^

Other radiological signs of aggressiveness are: location between Th3 and Th9, involvement of the entire vertebral body, extension to the neural arch, expanded cortex with indistinct margins, irregular honeycomb pattern, and soft-tissue mass.^7^

Determination of aggressiveness is an important part of imaging evaluation, as it influences the decision about the treatment. According to Deramond, considering radiological signs of aggressiveness, VH can be classified into four groups:

1. Asymptomatic VH, without radiological signs;
2. Asymptomatic VH, with radiological signs;
3. Symptomatic VH, without radiological signs;
4. Symptomatic VH, with radiological signs (13).

Patients in group 1 require no treatment. Patient from all other groups need some kind of intervention.^15^

Aggressive VH could closely resemble solitary plasmacytoma. Lytic appearance on CT is characteristic for both lesions. Low signal on T1 and high signal on T2 images as well as marked enhancement on MR are also common features. A characteristic “mini brain” appearance on MR images is described to be a typical finding in plasmacytoma. Rare vertebral epitheloid haemangioendothelioma is another lytic lesion that could be very similar to aggressive haemangioma on CT and MR imaging.

Our patient presented with symptomatic VH like lesion that showed some radiological characteristics of aggressiveness (low fat content on standard MR imaging, extension to the neural arch, and expanded cortex with indistinct margins). Therefore the lesion could be classified into group 4 (according to Deramond).^15^

However, fat content, as important factor that determines VH and its behaviour (aggressiveness), could be evaluated only qualitatively on standard MR images. High signal on STIR sequence and particularly low signal on T1 sequence are features of aggressive VH. More accurate quantitative assessment of fat content could be very useful in some cases. This is especially important with lesions that do not present with typical imaging characteristics of haemangioma and in those haemangioma with some characteristics of aggressiveness. The later was true in our case.

MRS enables quantitative evaluation of biological tissue. Namely, the method has the ability to transform bulk MR imaging data (derived from examined tissue) to distinct components. Technique applied in our case (proton MR spectroscopy) uses the signal from hydrogen protons to determine the concentration of metabolites. Bone marrow is composed mainly of water and lipid. Accurate determination of these two key fractions can be achieved with spectral analysis using MRS.^10^,^16^ The method was used early to determine vertebral fat content in healthy individuals.^9^,^10^

We were able to perform a MRS in our patient and on the basis of collected data a spectral analysis of two vertebral bodies (affected and normal adjacent) was performed. MRS data from healthy lumbar vertebral body showed high lipid peak (high fat content) as expected for the patient’s age (68%FF). Our data were in concordance with data published by other authors.^17^ The analysis of age differences in the proton spectrum of vertebral bone marrow showed an increase in %FF with increasing age, from 24% in the age group of 11 to 20 years to 54% in the group aged 61 years or older.^17^ Specter from pathological lesion within the 12^th^ thoracic body was completely different, as expected. The water content was comparable to healthy vertebral body, while fat content was low, but still very obvious (33%FF).

There is another fact that must be considered analysing water content in vertebral bodies. It has been shown that the water fraction approximates the percentage volume of haematopoietic (red) marrow. It declined with age from 81–89% in the 2^nd^ decade to 37–45% in subjects older than 70 years.^10^ A hypothetic presence of a large amount of haematopoietic marrow (high water content) in the affected vertebra would result in a low %FF. According to our knowledge there are only two available reports of using vertebral body MRS in patients with multiple myeloma.^18^,^19^ The authors reported about decreased fat content in multiple myeloma (20% *vs*. 31% and 34% in volunteers and osteoporosis).^17^

Based on our low but still significant amount of %FF (33%FF), we could speculate that a haematopoietic origin of the lesion (*i.e*. solitary plasmacytoma) was less likely. The result of MRS could therefore confirm the diagnosis of aggressive haemangioma.

A dilemma about the treatment modality and time to intervention rose. Due to the size of the lesion, there was an obviously need for stabilization to avoid collapse of the vertebra. A minimally invasive percutaneous vertebroplasty with biopsy at the same time was chosen. The procedure was successfully done and the biopsy confirmed the diagnosis of aggressive haemangioma.

## Conclusions

The presented case shows that MRS can be used as an additional tool for diagnosis and evaluation of aggressiveness of vertebral haemangioma and haemangioma like lesions.
